# Metastatic rectal neuroendocrine carcinoma presenting with treatment-refractory immune thrombocytopenia: A case report and literature review

**DOI:** 10.1097/MD.0000000000029616

**Published:** 2022-07-22

**Authors:** Wouhabe Marai Bancheno, Sneha Rao Adidam, Mekdem Abiy Melaku

**Affiliations:** a Internal Medicine, Howard University Hospital, Washington, DC.

**Keywords:** bone marrow metastasis, immune thrombocytopenia, neuroendocrine carcinoma

## Abstract

Colorectal neuroendocrine cancer (CRNEC) accounts for < 1% of all colorectal cancers. It presents in advanced stages and carries a risk of poor survival. CRNEC may be diagnosed incidentally on imaging or routine endoscopy. When symptomatic, it may mimic colorectal adenocarcinoma. Syncope and immune thrombocytopenic purpura (ITP) have not previously been reported as clinical presentations.

**Patient concerns:**

A 57-year-old man with human immunodeficiency virus infection on treatment, seizure and stroke without residue presented for the evaluation of fall and syncope.

**Diagnosis:**

Physical examination revealed tachycardia, swelling, and ecchymosis of his proximal left lower extremity. Laboratory tests showed a new isolated thrombocytopenia of 26,000/mm^3^. Computed tomography for a trauma survey showed an incidental left posterior rectal wall mass. After hospital admission, his platelet count dropped to 14,000/mm^3^. A peripheral blood smear revealed low platelet count, no schistocytes or immature cells. ITP at a high risk for bleeding was diagnosed and treated with standard medical therapy but remained refractory. Bone marrow biopsy showed metastatic neuroendocrine carcinoma, likely from the rectum.

**Interventions:**

Patient received courses of high-dose dexamethasone and intravenous immunoglobulin. He also receive eleven units of platelet transfusion. A course of rituximab was administered. The platelet count response was suboptimal or short lived with drop to a nadir of 4000/mm^3^. However, after initiation of Eltrombopag, the thrombocytopenia resolved. Patient was started on etoposide, carboplatin, Atezolizumab. His hospital stay was complicated by neutropenia and sepsis, which was successfully treated.

**Outcomes:**

He was discharged to subacute rehab in stable condition. About 4 months later, he was readmitted for severe thrombocytopenia, septic shock, and acute respiratory failure. Despite appropriate treatment, the patient deteriorated and expired.

**Conclusion:**

CRNEC is a rare aggressive disease with dismal outcome that lacks standardized treatment. Metastasis to the bone marrow is uncommon and concomitant ITP has not been reported. We report a rare case of rectal neuroendocrine carcinoma metastatic to bone marrow associated with refractory ITP and review the relevant literature.

## 1. Introduction

Neuroendocrine cells are rare epithelial cells with endocrine functions that express neuronal markers and peptides. They are dispersed throughout the body, mostly in the gastrointestinal tract and lungs.^[1]^ A heterogeneous group of neuroendocrine neoplasia (NEN) arise from these cells. In the past, comparison of treatment outcomes of these neoplasms was difficult, partly due to an overlap in classification. The World Health Organization lately classified neuroendocrine neoplasia into 3 distinct categories namely neuroendocrine tumors (NET), high-grade neuroendocrine carcinoma (HGNEC), and mixed neuroendocrine neoplasia (MiNEN). NET are graded according to the extent of differentiation from low-grade to high-grade. HGNEC by definition is always poorly differentiated. MiNEN (sometimes referred to as collision tumor) is a tumor formed by a combination of neuroendocrine and non-neuroendocrine components. Neuroendocrine carcinomas demonstrate a high proliferation rate, as reflected by a high number of mitoses and a Ki-67 positive fraction of > 20%.^[2–4]^ Colorectal neuroendocrine carcinoma (CRNEC), which originates in the hind gut, is a rare aggressive cancer accounting for less than a percent of all colorectal cancer cases; however, in some studies that included collision tumor, this figure was as high as 3.9%.^[5–7]^ Patients with HGNEC may be asymptomatic and are often detected incidentally during imaging studies, endoscopy of the lower gastrointestinal tract, or biopsy. When symptoms are present, they may mimic those of colorectal adenocarcinoma.^[5,8–12]^ Currently, CRNEC is more frequently recognized and it occurs in the mean or median age range of 50 to 70 years in most studies.^[5,6,13–15]^ Owing to the lack of typical symptoms and aggressive behavior, over two-thirds of patients are diagnosed at an advanced stage, contributing to poor survival rates.^[5,6,14–16]^ Metastasis most commonly occurs in the liver, locally, and in regional lymph nodes. Bone marrow and brain metastasis are rare.^[8–10,15,17,18]^ CRNEC presenting with refractory thrombocytopenia has not been described previously. Treatment of CRNEC involves surgery, chemotherapy, or radiation in various combinations based on the extent of the local tumor burden and metastasis.^[19]^ Despite this, the response rate is short, and the median survival of CRNEC patients with metastasis is <1 year.^[7,14]^ In this article, we present the case of a 57-year-old who presented to the emergency room for syncope and fall, found to have severe treatment-refractory thrombocytopenia, a rectal mass, and a bone marrow biopsy result consistent with the diagnosis of high-grade small-cell neuroendocrine cancer, likely originating from the rectal mass. We discuss relevant literature on CRNEC, its metastasis to the bone marrow and associated thrombocytopenia.

## 2. Case Report

**Figure 1.**
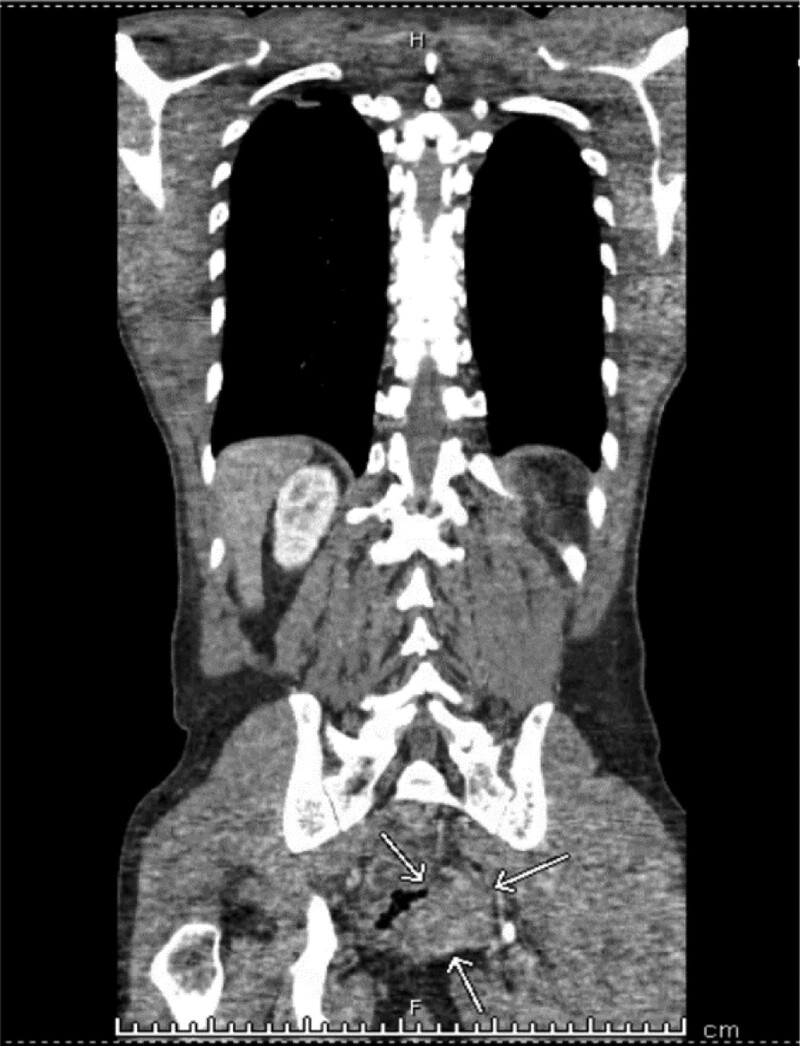
CT abdomen and pelvis with IV contrast showing 4.1 × 5.6 cm enhancing soft tissue mass in the left posterior wall of the rectum (thin white arrows).

**Figure 2.**
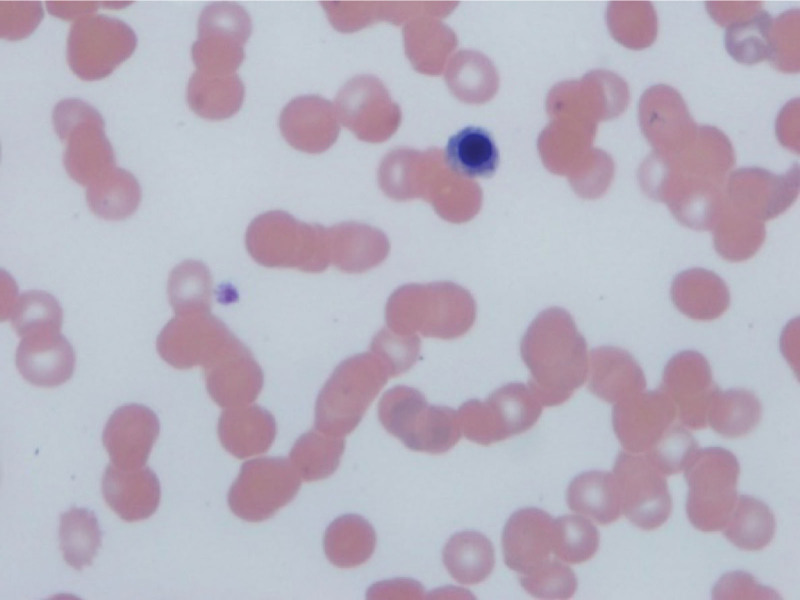
Peripheral smear showing nucleated red blood cells and only 1 platelet.

**Figure 3.**
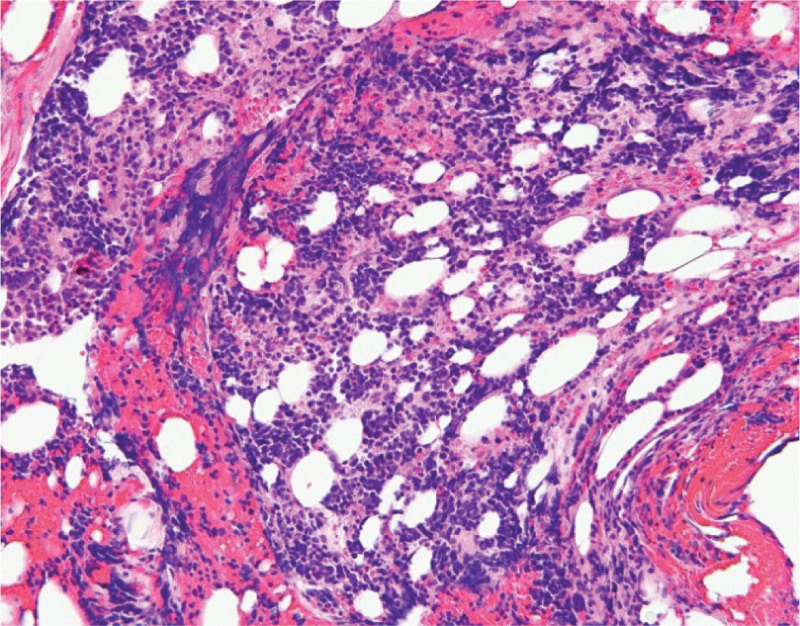
Bone marrow smear (at higher magnification) showing small round blue cells with areas of crushed cells, suggesting small-cell type neuroendocrine carcinoma.

**Figure 4.**
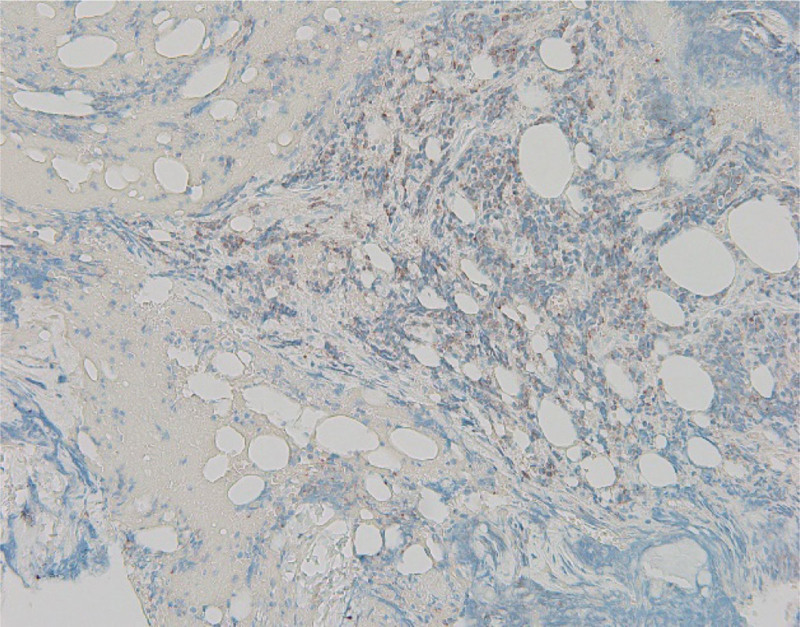
Bone marrow stain showing positive chromogranin and synaptophysin.

**Figure 5.**
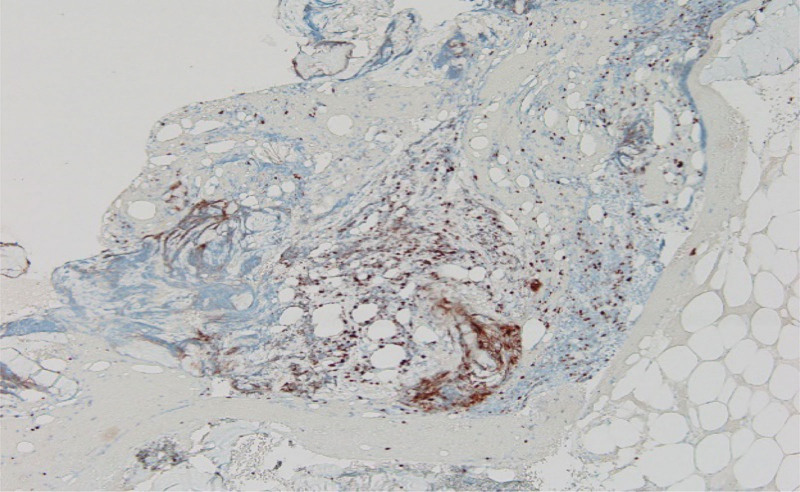
Bone marrow smear showing positive TTF-1 staining; synaptophysin and chromogranin are common neuroendocrine markers. TTF-1 may also be positive in neuroendocrine carcinomas. TTF-1 = thyroid transcription factor-1.

A 57-year-old man with stable human immunodeficiency virus (HIV) infection on treatment, seizure, stroke without residue presented to the emergency room for evaluation of a fall and syncope. He had mild lower back, neck, and arm pain but denied gastrointestinal or constitutional symptoms. Vital signs were stable except a brief episode of hypotension and tachycardia. He was well built, conversant, fully conscious, and ambulatory. The rest of the physical examination was benign, except for erythema, swelling, and ecchymosis on his proximal left lower extremity. He had a 2 × 1 cm left inguinal firm, mobile, and tender lymph node. He was trauma-triaged and cleared of a fracture or significant injury. On initial work-up, he was found to have thrombocytopenia of 26,000/mm^3^ compared to a platelet count of 170,000/mm^3^ a week ago. White blood cell count was 10,000/mm^3^, hemoglobin was 13 gm/dL, hematocrit was 38.4%, and chemistry was within the normal range. Urine toxicology result was positive for marijuana. Computed tomography of the chest, abdomen, and pelvis with intravenous contrast taken for trauma survey showed a left posterior rectal wall incidental mass contiguous with the sacrum without bony destruction or retroperitoneal lymphadenopathy. The mass measured 4.1 × 5.6 cm (Fig. 1). The patient was admitted to the internal medicine service for evaluation of syncope, rectal mass, thrombocytopenia, and recent trauma. Follow-up blood count showed a further drop in the platelet count to 14,000/mm^3^. A peripheral blood smear revealed reduced platelet count, no schistocytes, or immature cells (Fig. 2). Carcinoembryonic and prostate-specific antigen levels were not elevated. The diagnosis of immune thrombocytopenic purpura (ITP) with a high risk for bleeding was made after consulting with the hematology-oncology department. Two courses of high-dose dexamethasone and 3 doses of intravenous immunoglobulin were administered; however, the platelet count continued to drop below 10,000/mm^3^. Eleven units of platelet transfusions were required, but improvement in platelet count was never adequate or short lived. A course of rituximab was administered; however, the platelet count remained very low, reaching a nadir of 4000/mm^3^. After a course of Eltrombopag, the thrombocytopenia responded well, reaching the normal range. Bone marrow biopsy for treatment-refractory thrombocytopenia was performed by an interventional radiologist, which revealed mild hypercellularity and small-cell metastatic high-grade neuroendocrine cancer with Ki-67 > 90%, likely primary tumor in the rectum (Figs. 3–5). He was started on palliative chemotherapy of a combination of Etoposide and Carboplatin. During chemotherapy, he developed anemia, neutropenic fever, methicillin-resistant *Staphylococcus aureus* bacteremia, and sepsis likely source being pneumonia. He was treated with blood transfusion, Filgrastim, and intravenous antibiotics in the intensive care unit, and improved. Transesophageal echocardiogram was unremarkable. The patient was discharged to a subacute rehabilitation center in stable condition. Outpatient colonoscopy with endoscopic ultrasound confirmed the presence of a submucosal, nonobstructing rectal mass. Endoscopic biopsy revealed atypical cells. Resection of the rectal mass was deferred because of the advanced disease and the absence of local symptoms. He continued Etoposide, Carboplatin, and Atezolizumab as palliative chemotherapy as an outpatient. About 4 months after discharge, he was readmitted for severe thrombocytopenia, septic shock, and acute respiratory failure. Despite appropriate treatment, he deteriorated and expired.

## 3. Discussion

The classification of NEN for years was overlapping and confusing making comparison of outcome data difficult. Based on an improved understanding, the World Health Organization^[3,4]^ in 2019 published a new classification scheme comprising 3 entities of NEN of the gastrointestinal system: well-differentiated neuroendocrine tumors, poorly differentiated or high-grade neuroendocrine carcinoma, and mixed neuroendocrine and non-neuroendocrine neoplasia. The treatment patterns and prognoses for each category are relatively different. NET includes grades 1 to 3, based on the extent of cellular differentiation. HGNEC is no longer graded like NET. It is already of high grade by definition but separated into small- and large-cell types.^[4,20]^ Neoplasms with substantial neuroendocrine and non-neuroendocrine components are classified as MiNENs, which contain poorly differentiated adenocarcinoma component in the colorectum.^[2,4]^ The reported incidence and prevalence of gastrointestinal NEN has been increasing.^[21–23]^ Colorectal neuroendocrine cancers are rare aggressive malignancies with a poor prognosis. They account for < 1% of all colorectal cancers, but in some series, as high as 3.9% when MiNEN was coreported.^[5–7,24]^ In 1 report, the age-standardized incidence of CRNEC was approximately 2 per million person-years.^[25]^ The incidence of CRNEC has been increasing due to improved diagnostic pathology, imaging, and better classification, while adenocarcinoma of the colon and rectum has decreased during the same period of time.^[13,22,25]^ CRNEC, like neuroendocrine cancers in other locations of the body, expresses neuroendocrine markers synaptophysin, neuron-specific enolase, and chromogranin A, which help in the histologic diagnosis. They demonstrate a high proliferation rate, as reflected by a high number of mitoses with a Ki-67 positive fraction of > 20%. Neuroendocrine cancers are related to small-cell cancer of the lung and are similarly divided into small-cell type, which is the most common and large-cell type. The small-cell type is thought to have a worse prognosis, but the significance of such categorization for treatment reasons has not been conclusively established.^[6,15,16,25]^ Most patients present with advanced or late-stage disease due to the lack of specific symptoms and aggressive nature of the disease.^[16,20,26]^ The average or median age at the time of diagnosis ranges from 50 to 70 years in different studies.^[5–7,14,26–28]^ Our patient was 57 years of age and fell within this age range. As expected, the patient had metastatic disease that had spread to the bone marrow at the time of diagnosis. Conte et al reviewed 100 cases of CRNEC from the survival, epidemiology, and end result database in the period 1991 to 2013. The median age of the patients at diagnosis was 55 years. Approximately two-thirds of the cases were metastatic at the time of diagnosis.^[15]^ Alese et al retrospectively examined the treatment patterns and outcomes of all patients with HGNEC of the gastrointestinal system from a national cancer database and reported a mean age of 63 years. More than 75% of patients at diagnosis had stage III or IV disease.^[13]^ Bernick et al studied 38 patients with CRNEC, which were treated from 1975 to 1998 at a single institution, and he reported a mean age of 57 years. More than two-thirds of the patients had metastatic disease at diagnosis, mostly stage IV.^[6]^ Aytac et al reported 25 cases of high-grade NET of the colon, rectum, and anal canal treated from 1993 to 2011. They reported a mean age of 56.4 years and the majority of cases had metastasis at the time of diagnosis.^[5]^ Based on the 2004 to 2015 national cancer database, Fields et al^[14]^ also found that the median age at diagnosis of CRNEC patients was 65 years, and the majority of patients presented with stage IV disease. Of 126 patients with high-grade neuroendocrine carcinoma of the colorectum treated from 1991 to 2010, reported by Smith et al, the median age was 56 years, and 67% had advanced disease. In this study, anorectal neuroendocrine cancer was detected earlier than colonic neuroendocrine cancer because of pain and rectal bleeding. However, the authors concluded that early presentation of anorectal neuroendocrine cancer may not translate to better prognosis.^[16]^ Finally, Balasubramanyam et al studied 472 patients with small-cell neuroendocrine cancer of the colon and rectum from a national cancer database for treatment patterns and prognostic factors. The patients were treated from 2004 to 2013. More than two-thirds of the patients had extensive stage disease. The median patient age was 65 years. In conclusion, patients were diagnosed with colorectal neuroendocrine carcinoma in their twenties and nineties. The disease is recognized mostly in advanced stages, and this negatively affects prognosis.^[28]^

Patients with CRNEC, like ours, may be asymptomatic or have nonspecific symptoms at presentation. Diagnosis may be missed on histological examination and reported as adenocarcinoma or even carcinoid. Presentation may be similar to that of colonic adenocarcinoma and may include abdominal mass, rectal or abdominal pain, rectal bleeding, constipation, diarrhea, difficulty in defecation, anal discomfort, or stool caliber change. Constitutional symptoms such as weight loss, fatigue, fever, anorexia, or other symptoms such as nausea, vomiting, bowel obstruction, and hematemesis may be present but are less common.^[8–11,14,29–31]^ Syncope and severe thrombocytopenia, which were present in our patient, have not been previously reported. CRNEC may also be detected incidentally for the first time on a computerized demography performed for other reasons, such as trauma, as in our case^[12]^ or on routine endoscopy. Lack of early or specific symptoms and aggressive tumor behavior negatively affected survival, as seen in our case. Most CRNEC are not hormone-secreting and lack paraneoplastic syndromes of functioning NET. However, Cushingoid features have been described in CRNEC that metastasize to the liver.^[16]^

The diagnostic evaluation of CRNEC may include barium enema and lower gastrointestinal endoscopy with biopsy. Staging evaluation should include chest, abdominal, and pelvic computerized tomography with contrast and PET/CT. Preoperative magnetic resonance imaging and endorectal ultrasound have been used to evaluate local involvement of the rectum and anal canal wall.^[5,29]^ CRNEC and all neuroendocrine cancers generally metastasize to the liver, regional and distant lymph nodes, lungs, and pelvic peritoneum, causing bowel or ureteric obstruction.^[8–10,15,21,28]^ Bone metastasis can occur in HGNEC and patients may present with skeletal events such as bone pain, cord compression, pathological fractures, and hypercalcemia.^[27,32]^ Metastatic neoplasms in the bone marrow, including CRNEC, are rarely encountered in clinical practice, and the literature is limited to case reports such as ours. One study that looked at patient files spanning from 1995 to 2016 for metastatic neoplasms to the bone marrow found that the most common cancer spreading to the bone marrow was the breast, followed by the prostate, and both small and nonsmall-cell lung cancer. In this study, there were only 2 cases of neuroendocrine cancers out of 67 patients with bone marrow metastasis, indicating the rarity of neuroendocrine cancer metastasis to the bone marrow. In the same study, the majority of patients with bone marrow metastasis presented with an abnormal blood count prior to bone marrow evaluation.^[18]^ Neuroendocrine cancer with bone marrow infiltration from an unknown primary tumor with multiple liver metastases, presenting with anemia and thrombocytopenia has been described.^[33]^ Neuroendocrine carcinoma with bone marrow metastasis may present with pancytopenia, features of myeloproliferative disorders, or isolated thrombocytopenia, as in the present case. It has been suggested that thrombocytopenia should prompt bone marrow evaluation in the setting of unclear etiology or when unresponsive to therapy to search for undiagnosed malignancy, as in our patient.^[34]^

Thrombocytopenia in cancer patients is relatively common, and causes may include cytotoxic chemotherapy, other medications, radiation therapy, bone marrow infiltration or necrosis, disseminated intravascular clotting, thrombotic thrombocytopenic purpura, or secondary hematologic neoplasms. Isolated ITP has been reported to occur in association with solid cancers but rarely. This has not received much attention in the oncology literature.^[34,35]^ Similar to our case, it has been reported mostly in renal, lung, prostate, and breast cancers. The cause-effect relationship has not yet been established.^[36]^ In a review of case reports, one-half of ITP cases occurred concurrently with the cancer, 25% prior to cancer, and in the rest, sometimes after diagnosis and treatment of the cancer as a sign of recurrence or other causes. Most patients respond to steroid treatment, but only a few respond completely after surgical removal or chemotherapy of the underlying cancer. In 1 case, ITP occurred prior to cancer and remitted after surgical resection; long-lasting complete remission of ITP was ascribed to cancer resection.^[35]^ There has been a documented case report of refractory ITP suspected to be a paraneoplastic syndrome of a kidney tumor in which ITP failed to respond to standard therapy, including steroids, immunoglobulins, romiplostim, emergent splenectomy, rituximab, and eltrombopag. However, 1 year after nephrectomy, thrombocytopenia resolved, and autoantibodies were no longer detectable in the blood. Based on immunological studies, the authors proposed that antigenic mimicry is a cause of ITP.^[36]^ Some authors believe that in patients with ITP, an associated cancer must be considered; however, an extensive search for cancer is not warranted unless there is clinical suspicion.^[35]^ The severe thrombocytopenia in our patient required multiple platelet transfusions due to increased risk of bleeding. The patient did not respond to conventional treatment with intravenous immunoglobulin, high-dose steroids, and rituximab. However, the patient responded well to eltrombopag. HIV and antiretroviral therapy have been implicated in thrombocytopenia.^[37]^ Our patient was known to be HIV-positive on antiretroviral therapy for a long time. His HIV disease was asymptomatic, he tolerated antiretroviral medication, and his platelet count was normal a week prior to presentation. Therefore, HIV and antiretroviral medications were not believed to be the cause of the thrombocytopenia.

Treatment modalities for neuroendocrine carcinoma, including CRNEC, have developed from parallel experience in the management of small-cell lung cancer (SCLC) and to some extent from colorectal adenocarcinoma. Cisplatin and etoposide combination treatment has been developed as the standard treatment for SCLC. Because of SCLC similarities with extrapulmonary high-grade neuroendocrine cancer, the same treatment has been extended as the default treatment for high-grade neuroendocrine cancers, including CRNEC, since the 1990s.^[27,38]^ Based on retrospective data and expert opinions, a multidisciplinary treatment approach with chemotherapy, surgery, and radiation therapy yielded better responses in patients with CRNEC.^[15,19,28,38]^ According to the extent of spread, for the purpose of treatment, poorly differentiated neuroendocrine carcinoma outside of esophagus has been approached as localized or locoregional, locoregionally advanced or metastatic disease. Chemoradiation with platinum-based chemotherapy plus or minus sphincter-sparing abdominoperineal resection is recommended for the treatment of localized or locoregional disease of the anus and distal rectum. For sites other than the distal anorectum, radical surgery plus platinum-based systemic chemotherapy (often neoadjuvant) and, in some cases, triple therapy (resection, chemoradiation, and systemic chemotherapy) are considered appropriate options for patients who are at a high risk of recurrence.^[39,40]^ Locoregionally advanced disease with a large tumor bulk or positive nodes is treated with chemoradiation and systemic chemotherapy with or without resection (often after neoadjuvant chemotherapy). Distant metastases, as in our case, are treated with palliative platinum-based chemotherapy. For patients with distant metastatic disease, surgery has not been shown to improve prognosis and has not been recommended. Systemic chemotherapy with cisplatin or carboplatin, which has a lower toxicity and equivalent efficacy, plus etoposide (irinotecan with cisplatin optional) for at least 4 to 6 cycles, continued until maximal response, is recommended in all categories of CRNEC cases.^[9,14,19,25,27–29,38,41–44]^ Neoadjuvant chemotherapy has been advocated for the purpose of downstaging the cancer because of the concern for a relatively high proportion of positive surgical margins, to improve resection, and to lower the risk of systemic recurrence. Patients with early stage disease treated with surgical resection alone had inferior outcomes compared to those who received neoadjuvant or adjuvant chemotherapy, suggesting that micrometastases contributed to poor surgical outcomes. The response rate to the platinum-etoposide regimen is rapid and can range from 30 to 67% but remission is usually short lived with poor survival.^[9,13,15,45,46]^ Radiation therapy has been offered as part of the initial therapy or for palliation, and has been found to be effective in alleviating symptoms. Brain metastasis, unlike SCLC, is rare, and cranial irradiation is not recommended.^[16,47]^ The role of second-line therapy for CRNEC has been debated. If relapse occurs 3 to 6 months after the discontinuation of first-line chemotherapy, there is no established second-line therapy, although this is being studied. Both FOLFIRI (folinic acid, 5-fluorouracil, and irinotecan) and FOLFIRI plus oxaliplatin (FOLFIRINOX) have been reported to show some activity in this scenario.^[9,19,29,48]^ Improved knowledge of the molecular and cellular biology of neuroendocrine cancers may provide effective treatments in the future, including targeted gene and immune checkpoint inhibitor therapy. In patients with metastatic CRNEC with deficient mismatch repair (dMMR), high microsatellite instability (MSI-H), and BRAF mutation, addition of immune checkpoint inhibitor therapy is recommended.^[16,19,26,29,44,49]^ Our patient received such therapy but did not undergo a tumor cell molecular study.

The life span of our patient from diagnosis to death was 4 months. This short period was not surprising, as he presented with bone marrow metastasis at an advanced stage of the disease. The reported median survival of patients with CRNEC in the literature is similar, mostly < 12 months. Survival was generally worse for patients with metastatic disease, those who did not receive chemotherapy, and those who did not undergo surgical intervention for localized disease. Male sex, advanced age, and left-sided cancer have been linked to poor prognosis in some studies.^[6,7,14,15,24,25,28,50]^ Field and colleagues studied 1208 patients with CRNEC from a national cancer database registry spanning from 2004 to 2015 where most patients had advanced disease. The median age was 65 years; 64.4% of them received chemotherapy, 61.3% received some form of surgery, and 21.2% received radiation therapy. They found an overall median survival of 9 months with 5.9 months in the metastatic disease group and 18 months in the nonmetastatic disease group from the time of diagnosis. In the multivariate analysis, survival was worse in the elderly and in the presence of metastasis. However, better survival was noted in patients who received chemotherapy and surgery for rectal tumors and nonmetastatic disease. Radiation therapy, the type of which was not specified, did not improve survival.^[14]^ Smith et al retrospectively studied 126 colorectal HGNEC patients from a single-center majority with advanced disease. They found that the overall median survival was 13.2 months irrespective of the treatment modality. Multivariate analysis revealed that the absence of metastasis was associated with better survival. With metastasis, the chemotherapy response was the only factor associated with survival. They concluded that surgery, particularly in the presence of metastasis, may not offer a survival advantage.^[16]^ Conte et al studied 100 patients with high-grade CRNEC, with a median age of 55 years, from a single institution treated from 1991 to 2013. The majority of patients (64%) had advanced-stage disease and were treated with a multimodal approach depending on the extent of the disease. The median overall survival was 14.7 months, while that for metastatic disease was 8.7 months. On multivariate analysis, older age, advanced-stage disease, and high lactate dehydrogenase levels were associated with poor outcomes. In patients with localized disease, multimodal therapy was associated with a trend toward improved median overall survival.^[35]^ Patta and Fakih described a small series of 8 cases of high-grade metastatic NET of the colon and rectum, in which patients received cisplatin and etoposide chemotherapy from a single institution pharmacy and tumor registry for the period to 2003 to 2010. The median progression-free survival was 4.5 months, and the median overall survival was 9.5 months which also corroborated the poor prognosis of the disease despite treatment.^[27]^ Bernick et al studied 38 patients of colorectal NEC, from a single institution in the United States of America, seen from 1975 to 1998, with a mean age of 57 years. Most patients presented with advanced-stage disease and heterogeneous treatment with single or combination modalities, including surgery, chemotherapy, or RT, were offered. They reported an overall median survival of 10.4 months. Unlike other investigations,^[7,25]^ no difference in survival between large and small-cell types was found.^[6]^ A relatively old study by Saclarides (1994) and associates reported 39 cases of CRNEC, which by current definition included MiNEN cases with a mean age of 65.5 years. The mode of management of the patients was not clear; however, the median survival for patients with pure CRNEC was 7 months. The overall prognosis was poor in the metastatic group and small-cell-type pathology. Age was not associated with poor outcome.^[7]^ Balasubramanyam et al reported 472 cases of small-cell cancer of the colon from the National Cancer Database spanning from 2004 to 2013. More than two-thirds of the patients had extensive stage disease, and patients received monotherapy or combinations of surgery, chemotherapy, and radiation therapy. Chemotherapy has been noted to improve survival in both limited and extensive disease. However, contrary to consensus, surgery did not seem to improve survival in either group of patients in this study. Contrary to current beliefs, radiation therapy has been associated with improved survival in patients with extensive disease. The median survival for extensive disease in this study was 4 months, whereas for limited disease, it was longer.^[28]^ More recently, Chen et al analyzed 344 patients of colorectal neuroendocrine carcinoma. The majority of the patients had advanced disease and they were treated with radical surgery and chemotherapy. The overall median survival time of the patients was 9 months. In this study, radical surgery and chemotherapy were associated with improved survival.^[24]^

## 4. Conclusion

In conclusion, in this article we presented a rare case of rectal HGNEC metastasizing to the bone marrow that received diagnostic attention for incidentally diagnosed ITP refractory to initial therapy. We also discussed the relevant literature on CRNEC. CRNEC is an aggressive, rare disease that is increasingly being recognized and occurs mostly in the median or average age range of 50 to 70 years. It has been reported in patients as young as in 20s and as old as in 90s. Symptoms and signs of CRNEC are indistinguishable from those of colorectal adenocarcinoma. CRNEC also can be asymptomatic and detected incidentally on imaging or endoscopy. CRNEC metastasizes locally and distant structures, mostly to the liver. Brain and bone marrow metastases are rare. The occurrence of severe refractory ITP has not been described in association with CRNEC but could occur with other solid tumors. Currently, CRNEC is treated with surgery, chemotherapy, radiation therapy, and various combinations, depending on its location, tumor burden, and presence of metastasis. Despite treatment, the overall prognosis is poor, and the median survival for patients with metastatic or extensive disease is barely a year. Owing to the rare nature of the disease, patients are better treated in high-volume centralized facilities with a multidisciplinary approach. Prospective studies involving multiple centers may be challenging to conduct because of the rarity of this disease. However, further studies are required to generate evidence for the optimal effectiveness of various treatment modalities in patients with CRNEC.

## Acknowledgments

We would like to acknowledge department of pathology, Howard University Hospital for providing annotated slides. We would like to thank the department of radiology, Howard University Hospital for sharing the CT scan image in this article. Last but not list we also would like to extend our thanks to interdepartmental consultation team for participating in the care of the index patient.

## Author contributions

Sneha Rao Adidam drafted the case report. Wouhabe Marai Bancheno and Mekdem Abiy Melaku searched the literature, selected and reviewed the articles. The team drafted, edited and finalized the article.
